# Relationship of Mitochondrial-Related Protein Expression with the Differentiation, Metastasis, and Poor Prognosis of Oral Squamous Cell Carcinoma

**DOI:** 10.3390/cancers15164071

**Published:** 2023-08-11

**Authors:** Aki Murakami, Daisuke Takeda, Junya Hirota, Izumi Saito, Rika Amano-Iga, Nanae Yatagai, Satomi Arimoto, Yasumasa Kakei, Masaya Akashi, Takumi Hasegawa

**Affiliations:** Department of Oral and Maxillofacial Surgery, Kobe University Graduate School of Medicine, Kobe 650-0017, Japan; aki0221@med.kobe-u.ac.jp (A.M.); dsktkd@med.kobe-u.ac.jp (D.T.); arinco@med.kobe-u.ac.jp (S.A.); ykakei@med.kobe-u.ac.jp (Y.K.); akashim@med.kobe-u.ac.jp (M.A.)

**Keywords:** mitochondrial dysfunction, mitochondrial tumor-suppressor protein, mtDNA-repair protein, oral squamous cell carcinoma

## Abstract

**Simple Summary:**

Mitochondrial dysfunction and respiratory function changes have been associated with the initiation and progression of cancer. However, no study has comprehensively investigated the relationship between these mitochondrial-related factors and prognosis in a large number of patients with oral squamous cell carcinoma (OSCC). Here, we retrospectively investigated the expression of mitochondrial tumor-suppressor and DNA-repair proteins (PGC-1α, TFAM, OGG1, MTUS1, and SIRT3) in patients with OSCC and evaluated the relationship between their expression and prognosis. The expression levels of the five proteins were associated with patient outcomes. The 3-year disease-specific survival (DSS) rates of patients showing positive expression of all selected proteins were significantly higher than those of patients showing a lack of expression. Particularly, based on the results of multivariate analysis, negative expression of PGC-1α is related to a poor prognosis of OSCC. Low PGC-1α expression and vascular invasion may be clinically effective predictors of oral cancer prognosis.

**Abstract:**

Mitochondrial dysfunction and respiratory function changes have been consistently associated with the initiation and progression of cancer. The purpose of this study was to retrospectively investigate the expression of mitochondrial tumor-suppressor and DNA-repair proteins in patients with oral squamous cell carcinoma (OSCC) and to evaluate the relationship between their expression and prognosis. We enrolled 197 patients with OSCC who underwent surgical resection between August 2013 and October 2018. Clinical, pathological, and epidemiological data were retrospectively collected from hospital records. The expression of peroxisome proliferator-activated receptor gamma coactivator-1α (PGC-1α), mitochondrial transcription factor A, mitochondrial tumor suppressor gene 1, silent information regulator 3, and 8-hydroxyguanine DNA glycosylase was investigated using immunochemistry. The 3-year disease-specific survival (DSS) rates of patients showing positive expression of all selected proteins were significantly higher than those of patients showing a lack of expression. Multivariate analysis revealed that the expression of PGC-1α (hazard ratio, 4.684) and vascular invasion (hazard ratio, 5.690) can predict the DSS rate (*p* < 0.001). Low PGC-1α expression and vascular invasion are potential clinically effective predictors of the prognosis of OSCC.

## 1. Introduction

Oral squamous cell carcinoma (OSCC) accounts for approximately 40% of head and neck squamous cell carcinoma (HNSCC) cases. Approximately 300,000 new OSCC cases are recorded worldwide each year, and the number continues to increase. Despite advances in systemic therapies such as chemotherapy and radiotherapy, as well as in surgery, the 5-year survival rate of patients with OSCC has not improved over the past 40 years [1,2,3].

Mitochondria are called the powerhouse of the cell because they fulfill most of the cellular energy requirements [4]. Adenosine triphosphate (ATP) generation depends on oxidative phosphorylation, which involves mitochondrial DNA (mtDNA). mtDNA is susceptible to damage; the mutagenesis rate of mtDNA is 10–20 times higher than that of the nuclear genome [5,6,7]. Other functions of mitochondria besides energy production have been reported; these include apoptosis induction, reactive oxygen species (ROS) generation, mitochondrial fission, and mitophagy [8].

Mitochondrial dysfunction and respiratory function changes have been consistently associated with the initiation and progression of cancer. Various types of solid tumors and blood-related malignancies exhibit mitochondrial abnormalities, and disrupted mitochondrial function has been implicated in aging, diabetes, neurodegenerative disorders, and muscle wasting [9]. Numerous genes are involved in these processes. Peroxisome proliferator-activated receptor gamma coactivator-1α (PGC-1α) is responsible for promoting mitochondrial biogenesis and respiration. It activates nuclear transcription factors, leading to the transcription of nuclear-encoded mitochondrial transcription factor A (*TFAM*). TFAM, in turn, regulates the transcription of genes in the mitochondria [10]. In our previous study, we investigated mtDNA copy number and PGC-1α and TFAM expression in normal and OSCC tissues and demonstrated that the PGC-1α–TFAM mitochondrial pathway may be inhibited in OSCC tissues [9,10].

The association between cancer and several other mitochondrial-related proteins, such as silent information regulator 3 (SIRT3), mitochondrial tumor suppressor gene 1 (MTUS1), and 8-hydroxyguanine DNA glycosylase (OGG1), has been reported [11,12,13,14,15,16,17,18,19,20,21,22]. *SIRT3* is an important gene that maintains mitochondrial redox balance; it encodes a primary mitochondrial deacetylase that interacts with at least one of the known subunits of complex I and reduces the formation of excess ROS [11,12]. PGC-1α acts as a transcription factor for *SIRT3* [13]. *MTUS1* is localized to 8p22, a chromosomal region that is frequently deleted in tumors [14]. At an advanced stage, oral cancer and head and neck cancer are characterized by reduced levels of mitochondrial tumor-suppressor proteins, including SIRT3 and MTUS1 [15,16]. In addition, OGG1 plays a crucial role in effective DNA damage repair and is encoded by a gene at 3p26.2, a region in the human chromosome, commonly associated with loss of heterozygosity in various human cancers [17,18]. Low OGG1 activity is associated with aggressive characteristics and prognosis of head and neck cancer, as well as other types of cancer [19,20,21,22]. However, to the best of our knowledge, no study has comprehensively evaluated the relationship between these mitochondrial-related factors and the prognosis of OSCC in a large number of patients. We hypothesized that if these mitochondrial-related factors are associated with the prognosis of oral cancer, they could be potential biomarkers for OSCC prognosis.

In this study, we retrospectively investigated the expression of mitochondrial-tumor suppressor (*PGC-1α*, *TFAM*, *MTUS1*, and *SIRT3*) and DNA-repair (*OGG1*) genes in patients with OSCC using immunochemistry and evaluated the relationship between their expression and prognosis of OSCC.

## 2. Patients and Methods

We enrolled 197 patients with OSCC who underwent surgical resection at the Department of Oral and Maxillofacial Surgery, Kobe University Hospital, Kobe, Japan, between August 2013 and October 2018. Clinical, pathological, and epidemiological data of the enrolled patients were retrospectively gathered from hospital records. Clinicopathological data, including details of age, sex, tobacco use, alcohol consumption, performance status (PS), subsite, clinical T classification (UICC/AJCC staging system 8th edition), histologic features (differentiation, vascular invasion, nerve invasion, and lymphatic invasion), pathological node status (extranodal extension (ENE) and multiple neck metastasis (MLM)), and treatment outcome, were investigated. All patients received clinical treatment according to the consensus guidelines for head and neck cancer [23]. We included patients who were diagnosed with squamous cell carcinoma and underwent radical surgery. Patients who had received chemotherapy and/or radiotherapy prior to the surgery were excluded from the study. This study was approved by the Ethics Committee of Kobe University Hospital (authorization number: 220192). As this was a retrospective study, obtaining informed consent from patients was not required. However, patients were provided the opportunity to refuse the use of their samples in this study.

Immunohistochemistry was performed on 4-μm sections of formalin-fixed, paraffin-embedded tumor specimens prepared in our pathology department. The sections were de-paraffinized with xylene, rehydrated through a graded alcohol series, and washed three times with phosphate-buffered saline (PBS). Heat-mediated antigen retrieval was performed in an ethylenediaminetetraacetic acid (EDTA) buffer of pH 9 (Dako, Carpinteria, CA, USA) in a water bath for 30 min. Endogenous peroxidase activity was inhibited by incubating the sections with 3% H_2_O_2_. The sections were then incubated overnight at 4 °C with appropriate dilutions of the following primary antibodies in Can Get Signal Immuno-stain Solution A (Toyobo, Osaka, Japan): rabbit polyclonal anti-PGC-1 alpha-N-terminal (1:500; ab191831; Abcam, Cambridge, UK), rabbit monoclonal anti-TFAM (1:100; ab176558; Abcam), rabbit polyclonal anti-OGG1 (1:100; NB100-106; Novus Biologicals, USA), rabbit polyclonal anti-MTUS1 (1:40; ab198176; Abcam), or rabbit monoclonal anti-SIRT3 (1:500; ab217319; Abcam). The sections were subsequently incubated with a horseradish peroxidase (HRP)-conjugated anti-rabbit IgG polyclonal antibody (#424142; Nichirei Bioscience, Tokyo, Japan) for 1 h at room temperature (22–25 °C) without diluting. The signal was developed as a brown reaction product by incubating with the peroxidase substrate 3,3-diaminobenzidine (#415171; Nichirei Bioscience) for 10 min at room temperature. The sections were counterstained with hematoxylin and observed at ×200 magnification under a BZ-X800 microscope (Keyence, Osaka, Japan). The expression of each protein was determined independently and scored using the BZ-H3C/Hybrid cell count (Keyence). The most invasive part of the tumor was observed, and its location was standardized using the expression of the five proteins.

We evaluated the discriminatory ability of the expression of the five proteins (positive vs. negative expression) as an indicator of disease-specific survival (DSS) using a receiver operating characteristic (ROC) curve to determine the cutoff values for clinical tests. The area under the curve (AUC) ranged from 0.5 to 1, and it was used to measure the accuracy of this discrimination. The cutoff values were selected to minimize false positives and false negatives. The cutoff values for negative and positive expressions of the five proteins were determined using the ROC curve method. The cutoff values for PGC-1α, TFAM, OGG1, MTUS1, and SIRT3 were 18.27%, 37.96%, 22.51%, 32.00%, and 26.16%, respectively (Table 1).

All statistical analyses were performed using SPSS 22.0 (IBM, Armonk, NY, USA) and Ekuseru-Toukei 2012 (Social Survey Research Information Co., Ltd., Tokyo, Japan) software. The association of each variable with mitochondrial-related proteins was analyzed using Fisher’s exact test for categorical variables. Cumulative overall survival (OS), disease-specific survival (DSS), distant metastasis (DM), regional control (RC), and local control (LC) rates were calculated using the Kaplan–Meier product limit method. The LC, RC, and DM were measured from the date of surgery to the date of first recurrence (local, regional, or distant), respectively, or the last follow-up. DSS was measured from the date of surgery to the date of death or the last follow-up, and the data of patients who died of causes other than OSCC were censored at the time of death. The significance of the curves was determined using the log-rank test. Results with *p* < 0.05 were considered significant. ENE and MLM were excluded from the multivariate analysis because they are strong prognostic factors that can be determined only after neck dissection. Therefore, the association between preoperative variables, including the expression of the five proteins, and DSS was introduced in multivariate Cox proportional hazards models. Forward stepwise algorithms were used to reject the variables that did not fit the model significantly. The hazard ratio (HR) and 95% confidence interval (CI) were determined.

## 3. Results

The clinical characteristics of the patients with OSCC are summarized in Table 2. Among 197 patients, 106 were male (53.8%) and 91 were female patients (46.2%), and their mean age was 67.5 ± 13.0 (range = 15–89) years. The most common primary tumor site was the tongue (92 patients; 46.7%), followed by the lower gingiva (43 patients; 21.9%), upper gingiva (30 patients; 15.2%), and buccal mucosa and floor of the mouth (16 patients; 8.1%). The T classifications were T1 in 36 (18.3%), T2 in 66 (33.5%), T3 in 30 (15.2%), and T4a and 4b in 65 (33.0%) patients (Table 2).

**Figure 1:**
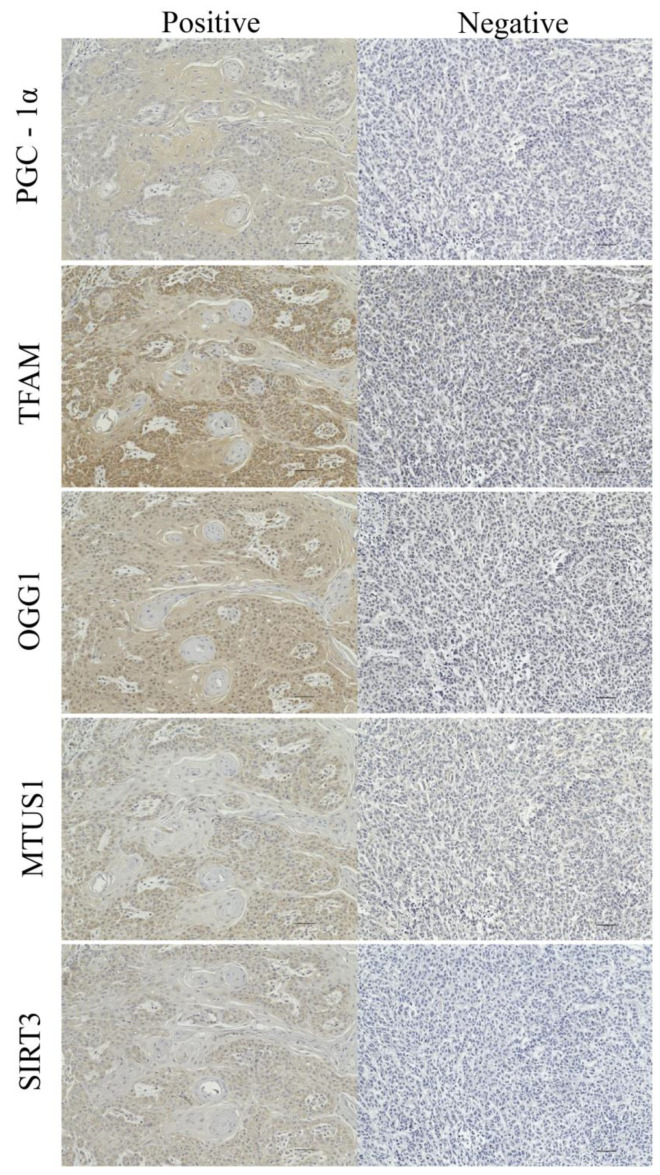
Expression analysis of the five selected proteins using immunohistochemistry. Immunostaining showed that PGC-1α, TFAM, OGG1, MTUS1, and SIRT3 are almost entirely localized to the cytoplasm. The images are shown at ×200 magnification. Scale bar = 50 μm.

Immunostaining showed that all five proteins (PGC-1α, TFAM, OGG1, MTUS1, SIRT3) were localized almost entirely to the cytoplasm. The expression levels of the five proteins are shown in Figure 1.

We investigated the association between the expression of the five proteins and clinicopathological factors (Table 3). 

No significant relationships were observed between protein expression and the clinicopathological parameters of age, sex, alcohol consumption, tobacco use, and PS. The expression of all proteins was significantly associated with the primary tumor site (tongue or otherwise), clinical T stage, clinical N stage, vascular invasion, nerve invasion, lymphatic invasion, and multiple neck metastases. The expression of all proteins was significantly higher in T1 and T2 stages, which are considered early clinical T stages. In addition, significant expression of all proteins was observed at clinical N stages 0 and 1. The expression of PGC-1α, TFAM, and OGG1 was significantly associated with ENE and was high in negative cases (*p* = 0.022, *p* = 0.013, and *p* = 0.044, respectively). The expression of PGC-1α and MTUS1 was significantly higher in patients with well-differentiated or moderately differentiated tumors than in those with poorly differentiated tumors (*p* = 0.014 and *p* = 0.018, respectively).

**Figure 2:**
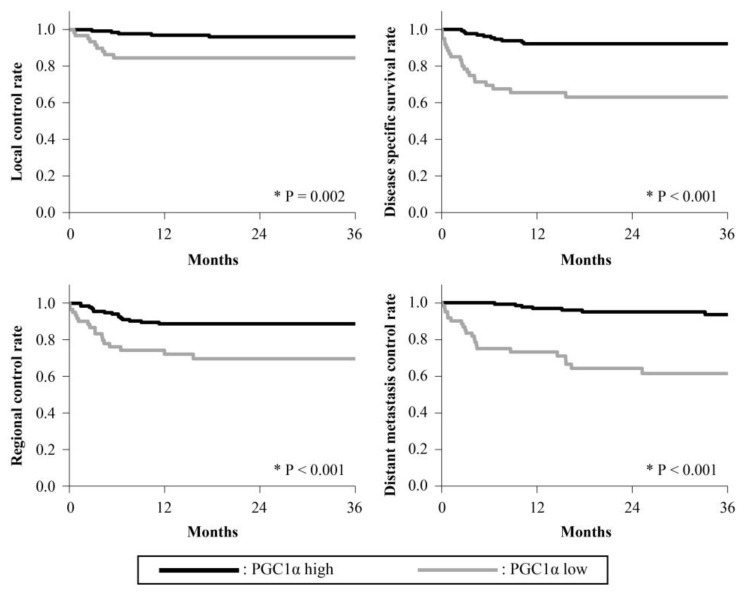
Cumulative 3-year survival rates of patients with high and low expression of PGC-1α. Cumulative 3-year local control (LC), disease-specific survival (DSS), regional control (RC), and distant metastasis control (DM) rates of patients with high and low expression of PGC-1α were 96.07% and 84.54%; 92.26% and 63.61%; 88.81% and 69.75%; and 93.58% and 61.64%, respectively. * Statistically significant (*p* < 0.05).

**Figure 3:**
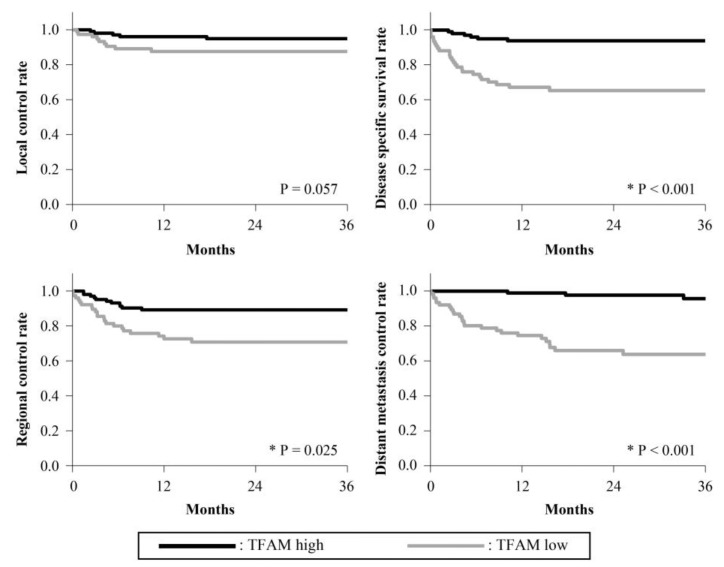
Cumulative 3-year survival rates of patients with high and low expression of TFAM. Cumulative 3-year local control (LC), disease-specific survival (DSS), regional control (RC), and distant metastasis control (DM) rates of patients with high and low expression of TFAM were 95.36% and 88.73%; 94.43% and 68.61%; 90.17% and 73.20%; and 96.15% and 67.06%, respectively. * Statistically significant (*p* < 0.05).

**Figure 4:**
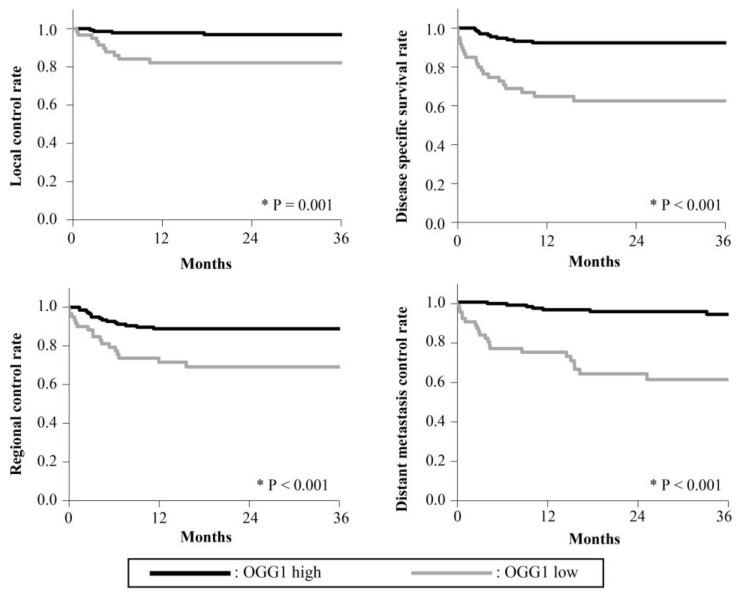
Cumulative 3-year survival rates of patients with high and low expression of OGG1. Cumulative 3-year local control (LC), disease-specific survival (DSS), regional control (RC), and distant metastasis control (DM) rates of patients with high and low expression of OGG1 were 96.86% and 82.14%; 92.40% and 62.50%; 88.90% and 69.18%; and 93.78% and 60.72%, respectively. * Statistically significant (*p* < 0.05).

**Figure 5:**
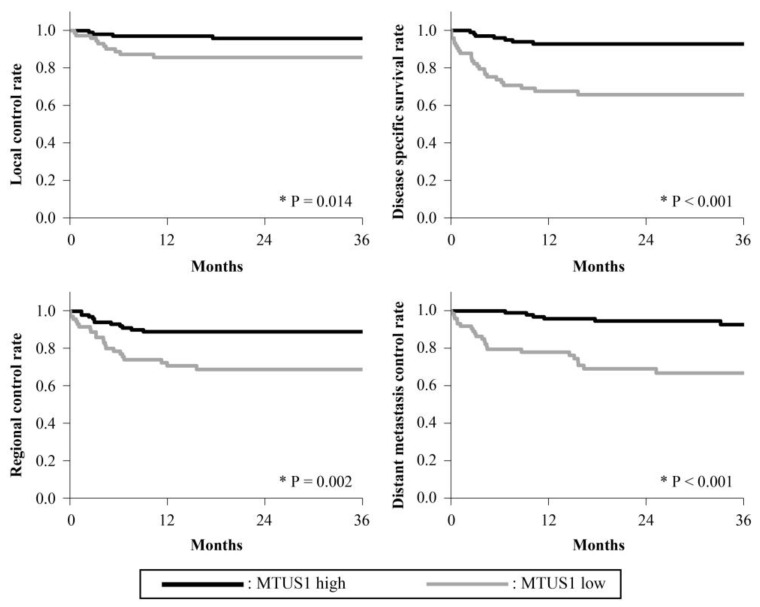
Cumulative 3-year survival rates of patients with high and low expression of MTUS1. Cumulative 3-year local control (LC), disease-specific survival (DSS), regional control (RC), and distant metastasis control (DM) rates of patients with high and low expression of MTUS1 were 96.28% and 87.15%; 93.56% and 69.17%; 90.32% and 72.60%; and 93.45% and 70.12%, respectively. * Statistically significant (*p* < 0.05).

**Figure 6:**
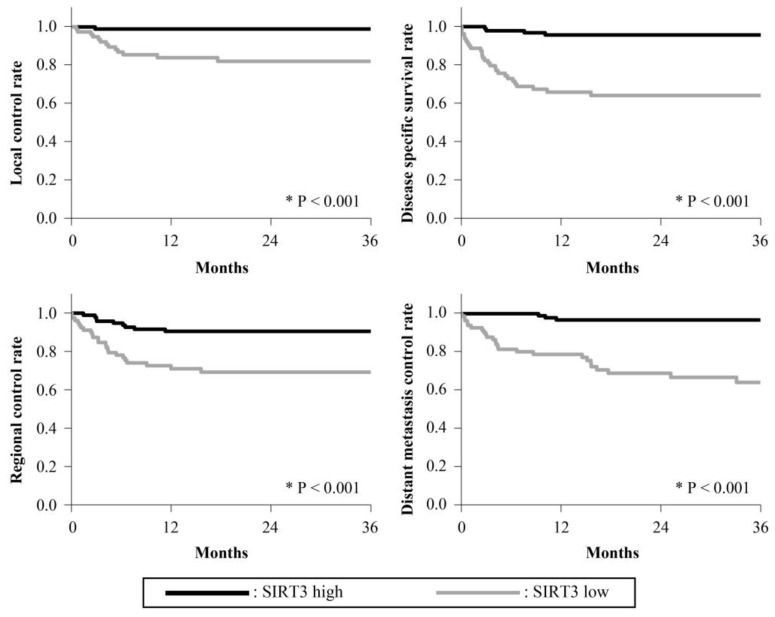
Cumulative 3-year survival rates of patients with high and low expression of SIRT3. Cumulative 3-year local control (LC), disease-specific survival (DSS), regional control (RC), and distant metastasis control (DM) rates of patients with high and low expression of SIRT3 were 99.07% and 84.14%; 96.10% and 67.83%, 91.53% and 72.54%; and 97.05% and 67.23%, respectively. * Statistically significant (*p* < 0.05).

The 3-year survival rates are presented in Figure 2, Figure 3, Figure 4, Figure 5 and Figure 6. The 3-year LC rates of patients with positive expression of four proteins, except TFAM, were significantly higher than those of patients with negative expression (PGC-1α: *p* = 0.002; OGG1 and MTUS1: *p* = 0.001; SIRT3: *p* < 0.001). The 3-year DSS rates of patients with positive expression of all proteins were significantly higher than those of patients exhibiting negative expression (*p* < 0.001). High expression of all proteins significantly correlated with increased RC rates compared with negative expression (PGC-1α, OGG1, and SIRT3: *p* < 0.001, TFAM: *p* = 0.025; MTUS1: *p* = 0.002). The DM control rates of patients with positive overall expression were higher than those of patients with negative expression. In the multivariate analysis, substantial PGC-1α expression (HR = 4.684) and vascular invasion (HR = 5.690) remained the most predictive factors of DSS (*p* < 0.001) (Table 4).

## 4. Discussion

The presence of mtDNA mutations is a crucial factor in the onset and progression of various cancers, including HNSCC [24,25]. Oxidative damages to mtDNA are restored by DNA-repair pathways. OGG1 is a DNA glycosylase that is important in the base excision repair pathway and is detected in the mitochondria [5]. Recent studies have indicated that OGG1 activity is regulated by SIRT3 and MTUS1. Similarly, decreased MTUS1 expression may be associated with advanced oral tongue SCC [15]. PGC-1α regulates mitochondrial biogenesis and cellular metabolism and activates SIRT3 [26]. Loss of SIRT3 expression increases the acetylation and degradation of OGG1, which ultimately increases ROS generation and carcinogenesis [27]. TFAM is required for the maintenance and biogenesis of mtDNA. It has been implicated in the growth and invasion of tumors [28,29]. In our previous study, we investigated mtDNA copy number and expression of PGC-1α and TFAM in normal and OSCC tissues and found that the PGC-1α–TFAM mitochondrial pathway might be inhibited in OSCC tissues [9]. Therefore, it is important to investigate the relationship between OSCC progression and mitochondrial-related protein expression considering that there are no effective targeted agents that substantially improve the prognosis of patients with OSCC. However, there is a lack of comprehensive evaluation of the relationship between the expression of these proteins and prognosis in a large number of patients with OSCC. Therefore, in this study, we examined the expression of mitochondrial tumor-suppressor and DNA-repair proteins in human OSCC specimens using immunohistochemistry and correlated the expression with prognosis.

The dysfunction or genetic abnormalities in mitochondrial tumor-suppressor proteins such as SIRT3 and MTUS1 lead to disturbances in mitochondrial energy metabolism, triggering cellular transformation and tumor development [30]. Ding et al. (2012) demonstrated that downregulation of MTUS1 expression is a common phenomenon during the progression of oral tongue SCC and is correlated with poor differentiation and enhanced proliferation [15]. In a study on head and neck cancer, the SIRT3 level was found to be markedly decreased in cancer tissues and was lower in advanced stages than in early stages [16]. In our study, significant associations were also found between the T classification and protein expression. Decreased expression of these proteins, including MTUS1, was observed in patients with advanced stages of cancers, such as T3 and T4. In addition, decreased expression of MTUS1 was observed in poorly differentiated tumors in OSCC, which is consistent with the findings of the previous studies described above. Paz-Elizur et al. (2003) demonstrated that decreased OGG1 activity is a major risk factor for lung cancer [19]. Gangwar et al. (2009) reported that OGG1 expression increased the risk of bladder cancer and hepatocellular carcinoma [20]. Sova et al. [21] reported that the OGG1 level is considerably reduced in invasive breast cancer and that it is associated with aggressive features such as a high grade, increased proliferation, and lymphatic invasion. They also suggested that OGG1 is an independent factor for poor prognosis [21]. It has also been reported that disease progression is faster in patients with head and neck cancer when OGG1 activity is low, which indicates that a low OGG1 activity is associated with an increased risk of head and neck cancer [22]. Our results suggest that low OGG1 expression may be associated with a poor prognosis of OSCC. 

The expression of TFAM in endometrial cancer is associated with tumor invasion and metastasis, including lymph node and distant metastasis, and TNM stage advancement [31]. In breast cancer, TFAM-positive patients have been reported to have a relatively poor clinical prognosis [32]. TFAM may be associated with the promotion of cancer cell growth and metastasis in bladder, esophageal, gastric, and colon cancers. It has been shown that in HNSCC, TFAM, and mtDNA expression is markedly decreased in tumors and correlates negatively with disease progression [33]. According to another study, increased TFAM expression in colorectal, endometrioid, pancreatic, and ovarian cancers is associated with an unfavorable prognosis with tumor metastasis [20]. Studies have shown that PGC-1α is a tumor suppressor and promotes metastasis in several cancers, including breast, hepatocellular, colorectal, endometrial, prostate, and pancreatic cancers, as well as in several models of melanoma. In addition, biphasic expression of PGC-1α has been observed in breast, melanoma, colorectal, and ovarian cancers. Low expression of PGC-1α is associated with worse outcomes in breast and liver cancers. The absence of expression of PGC-1α and TFAM has been reported in certain types of ovarian cancer [34,35,36,37]. 

In this study, the expression of these five proteins was significantly associated with the N classification and multiple neck metastases. In addition, decreased expression of PGC-1α, TFAM, and OGG1 was significantly associated with ENE and lymphatic invasion. To the best of our knowledge, there are no reports on the relationship between mitochondrial tumor-suppressor and DNA-repair proteins and cervical node metastasis in OSCC. Our results also indicate a significant association between the expression of PGC-1α, TFAM, OGG1, MTUS1, and SIRT3 and the 3-year RC rate of patients with OSCC. These results indicate that mitochondrial tumor-suppressor and DNA-repair proteins are associated with cervical lymph node metastasis in OSCC. However, these results may be confounding because they are closely related to advanced cancers with a high T classification or other confounding factors.

In this study, the 3-year LC rates of patients with high expression of four selected proteins, excluding TFAM, were significantly higher than those of patients with low expression. The 3-year DM control rate and DSS rate of patients with high expression of all five proteins were higher than those of patients exhibiting low expression. Moreover, the multivariate analysis revealed that PGC-1α expression and vascular invasion were the most important predictors of 3-year DSS. Vascular invasion and lymphatic invasion are predictors of DSS and OS in patients with OSCC [38,39,40,41]. The results for vascular invasion are consistent with those described in the abovementioned reports [38,39,40,41]. In particular, the results of the multivariate analysis showed that low expression of PGC-1α is associated with a poor prognosis of OSCC. Therefore, PGC-1α could be a potential biomarker for OSCC prognosis. These results may help develop a new integrative approach for screening and diagnosing patients with OSCC. Future studies should examine whether PGC-1α can be detected in small sections such as biopsy tissue samples. Furthermore, this study serves as a foundation for future studies to establish mitochondria tumor-suppressor and DNA-repair proteins as therapeutic targets in OSCC. 

This study has a few limitations. First, this study was retrospective in nature. Despite conducting multivariate analysis to minimize the effect of confounding factors as much as possible, it was not possible to entirely eliminate bias. Second, the association between the expression of each protein and poor prognosis of OSCC and its mechanism is unclear. Future studies should include appropriate treatment modalities. Further research is needed to elucidate this mechanism, conduct large prospective cohort studies, and assess the predictors of prognosis.

## 5. Conclusions

The expression levels of mitochondria tumor-suppressor and DNA-repair proteins (PGC-1α, TFAM, OGG1, MTUS1, and SIRT3) were examined in tumor samples from patients with OSCC to determine the association of these proteins with patient outcomes. The 3-year DM control and DSS rates of patients showing positive expression of all selected proteins were significantly higher than those of patients showing a lack of expression. Particularly, based on the results of the multivariate analysis, negative expression of PGC-1α was related to a poor prognosis of OSCC. Low PGC-1α expression and vascular invasion may be clinically effective predictors of oral cancer prognosis.

## Figures and Tables

**Table 1:** Cutoff values for the positive expression level of each factor.

	Positive Expression Level, %		
Factor	Negative	Cutoff Value ^a^	Positive
PGC-1α	≤	18.27	<
TFAM	≤	37.96	<
OGG1	≤	22.51	<
MTUS1	≤	32.00	<
SIRT3	≤	26.16	<

^a^ Cutoff values were determined using a receiving operating characteristic curve.

**Table 2:** Characteristics of the patients.

Characteristic	No. of Patients	(%)
**Age (years)**		
<70	97	49.2
≥70	100	50.8
**Sex**		
Male	106	53.8
Female	91	46.2
**Tobacco use**		
Smoker	59	29.9
Non-smoker	138	70.1
**Alcohol consumption**		
Drinker	77	39.1
Non-drinker	120	60.9
**PS**		
≤1	190	96.4
>2	7	3.6
**Primary tumor site**		
Upper gingiva	30	15.2
Lower gingiva	43	21.9
Buccal mucosa	16	8.1
Tongue	92	46.7
Oral floor	16	8.1
**T classification**		
1	36	18.3
2	66	33.5
3	30	15.2
4a/b	65	33.0
**N classification**		
0 or 1	154	78.2
2 or 3	43	21.8
**ENE**		
Positive	25	12.7
Negative	42	21.3
Non lymph node metastasis or non-neck dissection	130	66.0
**Multiple neck metastases**		
Positive	37	18.8
Negative	87	44.2
Non-neck dissection	73	37.0
**Margin**		
Positive	28	14.2
Negative	169	85.8
**Tumor differentiation**		
Well	109	55.4
Moderate	71	36.0
Poor	17	8.6
**Vascular invasion**		
Positive	61	31.0
Negative	136	69.0
**Nerve invasion**		
Positive	41	20.8
Negative	156	79.2
**Lymphatic invasion**		
Positive	41	20.8
Negative	156	79.2
**Disease control Status**		
Survival	153	77.7
Death of local failure	14	7.1
Death of regional failure	8	4.1
Death of distant metastasis	10	5.1
Death of other disease	12	6.1

ENE: extra nodal extension.

**Table 3:** Associations between the five factors and clinicopathological factors.

		PGC-1α Expression			TFAM Expression			OGG1 Expression			MTUS1 Expression			SIRT3 Expression		
	n	Negative	Positive	*p*-Value	Negative	Positive	*p*-Value	Negative	Positive	*p*-Value	Negative	Positive	*p*-Value	Negative	Positive	*p*-Value
**Age**				0.281			0.115			0.757			0.196			0.254
<70	100	27	73		37	63		29	71		37	63		41	59	
≥70	97	34	63		47	50		31	66		45	52		48	49	
**Gender**				0.219			0.665			0.279			0.664			0.254
Male	1106	37	69		47	59		36	70		46	60		52	54	
Female	91	24	67		37	54		24	67		36	55		37	54	
**Exposure to tobacco**				0.867			0.638			0.738			1.000			1.000
Smoker	59	19	40		27	32		19	40		25	34		27	32	
Non	1138	42	96		57	81		41	97		57	81		62	76	
**Exposure to alcohol**				0.346			0.557			0.875			0.459			1.000
Drinker	77	27	50		35	42		24	53		35	42		35	42	
Non	1120	34	86		49	71		36	84		47	73		54	66	
**PS**				0.679			1.000			1.000			0.702			0.703
0 or 1	1190	58	132		81	109		58	132		80	110		85	105	
More than 2	7	3	4		3	4		2	5		2	5		4	3	
**Primary tumor site**				0.001 *			0.001 *			0.005 *			<0.001 *			0.007 *
Tongue	92	18	74		27	65		19	73		26	66		32	60	
Otherwise	1105	43	62		57	48		41	64		56	49		57	48	
**T classification**				<0.001 *			<0.001 *			<0.001 *			<0.001 *			<0.001 *
T1, T2	1102	8	94		15	87		8	94		16	86		17	85	
T3, T4	95	53	42		69	26		52	43		66	29		72	23	
**N classification**				<0.001 *			<0.001 *			<0.001 *			<0.001 *			<0.001 *
0 or 1	1154	29	125		46	108		26	128		46	108		52	102	
>2	43	32	11		38	5		34	9		36	7		37	6	
**ENE**				0.022 *			0.013 *			0.044 *			0.064			0.056
Positive	25	18	7		22	3		18	7		20	5		21	4	
Negative	42	17	25		24	18		19	23		23	19		25	17	
**Vascular invasion**				<0.001 *			<0.001 *			<0.001 *			<0.001 *			<0.001 *
Positive	61	30	31		39	22		30	31		40	21		43	18	
Negative	1136	31	105		45	91		30	106		42	94		46	90	
**Nerve invasion**				0.008 *			<0.001 *			0.007 *			0.002 *			0.001 *
Positive	41	20	21		28	13		20	21		26	15		28	13	
Negative	1156	41	115		56	100		40	116		56	100		61	95	
**Lymphatic invasion**				0.008 *			0.032 *			0.055			0.007 *			0.004 *
Positive	41	20	21		24	17		18	23		25	16		27	14	
Negative	1156	41	115		60	96		42	114		57	99		62	94	
**Multiple neck metastases**				<0.001 *			<0.001 *			<0.001 *			0.003 *			<0.001 *
Positive	37	26	11		32	5		27	10		29	8		32	5	
Negative	87	29	58		42	45		29	58		42	45		47	40	
**Tumor differentiation**				0.014 *			0.072			0.051			0.018 *			0.125
Well, Moderate	180	51	129		73	107		51	129		70	110		78	102	
Poor	17	10	7		11	6		9	8		12	5		11	6	

* Statistically significant (*p* < 0.05). ENE: Extra nodal extension.

**Table 4:** Results of multivariate Cox proportional hazards model analysis of predictors of disease-specific survival.

			95% CI	
Variable	*p*-Value	Hazard Ratio	Lower	Upper
PGC-1α	<0.001 *	4.684	2.189	10.022
Vascular invasion	<0.001 *	5.690	2.598	12.464

* Statistically significant (*p* < 0.05). CI: confidence interval.

## Data Availability

All data generated or analyzed during this study are included in this published article. The data that support the findings of this study are available from the corresponding author upon reasonable request.
